# Dr. Charles Willard Hamlin

**Published:** 1897-11

**Authors:** 


DR. CHARLES WILLARD HAMLIN, of Middleville, died at St. Luke's hospital, Utica, October 6, 1897, aged 58 years. He was a son of Joseph S. Hamlin and was born at Holland Patent in 1839. He was educated first in the local schools, then at Whitestown seminary, and afterward at Albany, graduating from the normal school in 1861. He began the study of medicine in the office of Dr. D. A. Crane at Holland Patent, but soon afterward the civil war began and he enlisted in Captain Thorpe's company that was subsequently attached to the 57th regiment N. Y. V., raised in New York City. Soon afterward he was appointed hospital steward of the regiment and it was in this capacity that the writer, having been assigned to duty as surgeon in that regiment in the winter of 1863, first made his acquaintance. Mr. Hamlin was one of the most faithful and conscientious soldiers, and though serving with the medical department his duties often took him into extreme danger, but he was absolutely fearless under fire, frequently going to the front line of battle and even to the skirmish line to succor the wounded.

After the war he completed his medical collegiate course at Bellevue, graduating in the class of '66. Dr. Hamlin established himself in practice immediately at Middleville, where early he obtained the confidence of the community and his professional colleagues. He was a member of the Medical Society of the County of Herkimer, of the Medical Society of the State of New York, and represented the latter as an official delegate in the first Pan-American Medical Congress, at Washington, in 1893. He was married September 12, 1867, to Miss Dora A. Varney, and his widow with one son, Dr. Varney B. Hamlin, of Clinton, survive.

Dr. Hamlin was the eldest of a family of eleven children and the first to die. The last time the Hamlin family was reunited was on the occasion of the golden wedding of the parents, which was celebrated in Holland Patent, September 18, 1888. Mrs. Hamlin, the mother, died February 18, 1896. The brothers and sisters of the deceased are: Mrs. James T. Hall, Chicago; Edward E. Hamlin, Oriskany Falls; Mrs. Cornelia Gosnell, Kansas City; Eugene Hamlin, Floyd; Frederick W. Hamlin, Chicago; George T. Hamlin, Watertown; Miss Adelaide Hamlin, Holland Patent, and William W. Hamlin, Des Moines, Ia. Joseph S. Hamlin, father of this family, is in his 88th year and resides at Holland Patent.

It has fallen to few medical men in Central New York to attain more conspicuous eminence than did Charles Willard Hamlin, who died in the midst of a successful career, in the vigor of a well-ripened manhood, lamented by loving relatives, warm personal friends and numerous citizens.

In another place in this edition we publish the proceedings of the Medical Society of the County of Herkimer at its memorial meeting.

